# Confined Layer Slip Process in Nanolaminated Ag and Two Ag/Cu Nanolaminates

**DOI:** 10.3390/ma17020501

**Published:** 2024-01-20

**Authors:** Mahshad Fani, Wu-Rong Jian, Yanqing Su, Shuozhi Xu

**Affiliations:** 1 School of Aerospace and Mechanical Engineering, University of Oklahoma, Norman, OK 73019, USA; mahshad.fani-1@ou.edu; 2 Department of Mechanical Engineering, Stanford University, Stanford, CA 94305, USA; wurong@stanford.edu; 3 Department of Mechanical and Aerospace Engineering, Utah State University, Logan, UT 84322, USA; yanqing.su@usu.edu

**Keywords:** confined layer slip, nanolaminate, dislocation, interface, atomistic simulations

## Abstract

The exceptional strength of nanolaminates is attributed to the influence of their fine stratification on the movement of dislocations. Through atomistic simulations, the impact of interfacial structure on the dynamics of an edge dislocation, which is compelled to move within a nanoscale layer of a nanolaminate, is examined for three different nanolaminates. In this study, we model confined layer slip in three structures: nanolaminated Ag and two types of Ag/Cu nanolaminates. We find that the glide motion is jerky in the presence of incoherent interfaces characterized by distinct arrays of misfit dislocations. In addition, the glide planes exhibit varying levels of resistance to dislocation motion, where planes with intersection lines that coincide with misfit dislocation lines experience greater resistance than planes without such intersection lines.

## 1. Introduction

Nanolaminated metals have emerged as a prominent area of research primarily due to their remarkable properties. These materials not only have impressive strength and exceptional stability during mechanical deformation [1,2,3], but also demonstrate enhanced resistance to irradiation [4,5], fatigue [6], and shock [7] compared with traditional metals. Among those that have been investigated, the layers of nanolaminates can either consist of the same material, as seen in nanotwinned Cu [8,9] and nanotwinned Ag [10], or they can be made from different materials, such as Cu/Ag [11,12] and Cu/Nb [13,14,15] bimetal nanolaminates. Metals like Ag and Cu, as well as the nanolayers comprising them, have some specific features that make them important. General nanolayers have shown high bactericidal and antiviral activity, suggesting their potential applicability in creating respiratory protection equipment [16,17]. Ag/Cu nanolaminates have attracted attention due to their superior electrical conductivity [18]. They are utilized in creating high-performance magnet windings essential for high-field magnets and in designing electronic devices [19]. Additionally, these materials are employed in the development of components for electric railways, heat transfer systems, plasma-facing elements, and non-equilibrium systems [20,21].

Consequently, the movement of dislocations within these layers is predominantly governed by the interfaces between the two metals. This is rather than by interactions among dislocations within crystalline structures themselves. The behavior and strength of these nanolaminates are significantly influenced by bimetal interface properties. Multilayered metals’ plastic deformation mechanisms are influenced by the microstructure of the interface. This in turn impacts the initiation of dislocation formation at the interface [22,23,24]. The thickness of each nanolaminate layer, denoted by *L*, also plays a crucial role in dictating the movement of dislocations within the layer. Specifically, within the usual thickness range found in nanolaminates, which is 5 nm <L< 100 nm, dislocations are observed to move via a process known as confined layer slip (CLS) [25,26,27].

Molecular dynamics (MD) simulation has been utilized to explore the processes of deformation in nanolaminate materials at both atomic and nanometer scales [28,29,30]. Up until now, the role of interface structure in contributing to CLS has not been extensively studied. The Ag/Cu nanolaminate is a metallic multilayer known for its remarkable attributes, including impressive strength, excellent electronic conductivity, and exceptional thermal stability [31,32,33]. The properties of bi-metal interfaces in Ag/Cu play a crucial role in the design of radiation-resistant materials [34,35] and also have an effect on dislocation and resistance of interface gliding [36]. Past research has concentrated on exploring how dislocations or deformation twins interact with these interfaces within Ag/Cu nanolaminates [37,38]. The effect of interface structure and layer thickness on the mechanical properties and deformation behavior of Ag/Cu nanolaminates has been investigated [29]. The role of temperature on plastic deformation under tensile loading in Ag/Cu nanolaminates has been examined as well [28]. Due to the transmission of dislocations across the interfaces, the interfaces in Ag/Cu nanolaminates can undergo considerable roughening [29].

In this study, we employ MD simulations to examine how interface structure influences the resistance to CLS in nanolaminates composed of face-centered cubic (FCC) Cu and FCC Ag. The Ag/Cu nanolaminates, often fabricated through bulk-forming techniques, have been chosen as a model material. The interface structure of an Ag/Cu bi-crystalline arrangement with the specified orientations can be determined by the crystallographic relationship between the two grains. Incoherent interfaces are identified between Ag and Cu. To provide references, we also consider nanolaminated Ag, where a symmetric incoherent twin boundary (SITB) is present. Additionally, to better understand the role of a solid interface in this context, calculations of glide have been performed in two Ag single crystals.

## 2. Materials and Methods

Large-scale Atomic/Molecular Massively Parallel Simulator (LAMMPS) [39] is utilized for conducting atomistic simulations. We acquired all the atomic configurations presented in this paper utilizing OVITO, a visualization and analysis software for output data generated in atomistic simulations [40]. It has been widely recognized that embedded atom method (EAM) potentials are the most commonly used model to describe atomic bonding in metal systems [41]. These potentials are capable of reproducing elastic constants, stacking fault energies, and vacancy formation energies [42], which are essential for simulating defect processes in strained crystals. In this study, the atomic interactions between Ag and Cu were characterized using the EAM potential developed by Williams et al. [43], which has been shown to well describe the Ag/Cu interface [28,29,37], the dislocation in Ag [44], and the dislocation in Cu [45].

### 2.1. Single Crystal Model

We consider the dislocation glide in an Ag single crystal to compare the results with those in nanolaminates. Two single crystal models are constructed: the first contains a relaxed, infinitely long edge dislocation (referred to as SC-Ag), while the second one contains a relaxed edge dislocation pinned between two {112} free surfaces (designated as FS-Ag). The SC-Ag model imposes traction-free boundary conditions along the *z* axis, whereas periodic boundary conditions (PBCs) are applied along the *x* and *y* axes. In the FS-Ag model, PBCs are applied along the *x*-axis, while traction-free boundaries are considered along the *y* and *z* axes. Likewise, the lattice coordinates and the dimensions of these two models are the same as those of grain *M*, i.e., [1 $\bar{1}$ 0]-[11 $\bar{2}$ ]-[111] and 43×5×26 nm^3^, respectively. The density functional theory-based {112} surface energies in Ag and Cu are 868 mJ/m^2^ and 1626 mJ/m^2^, respectively [46], while our model predicts a surface energy of 991.42 mJ/m^2^ for the same surface in Ag.

### 2.2. Bicrystal Model

**Figure 1:**
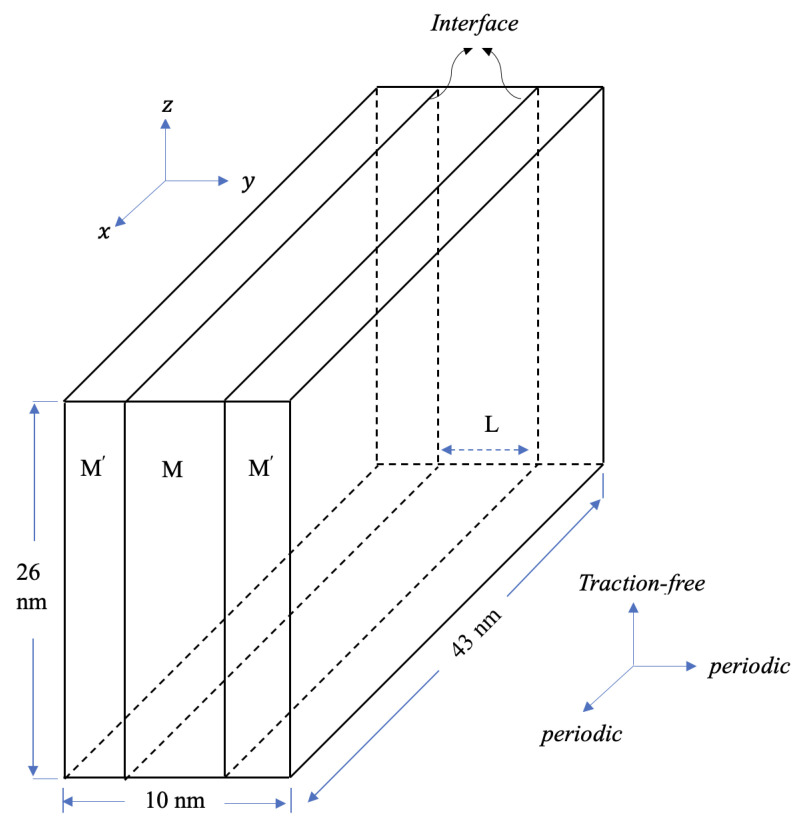
Schematics of the atomic configuration used to simulate CLS in the nanolaminates. *M* and $M^{\prime }$ consist of two single crystalline layers that are different in orientation and material in some cases. There is an edge dislocation inserted in the middle layer *M*.

The single crystal layers *M* and $M^{\prime }$ share identical layer thicknesses, each measuring *L* = 5 nm as shown in Figure 1. In these layers, the *x*, *y*, and *z* axes denote coordinates for layer *M*, while $x^{\prime }$ , $y^{\prime }$ , and $z^{\prime }$ pertain to layer $M^{\prime }$ . We have applied PBCs along the *x* (or $x^{\prime }$ ) and *y* (or $y^{\prime }$ ) axes, while traction-free boundary conditions are implemented along the *z* (or $z^{\prime }$ ) axis [14,47,48,49]. The crystallographic orientations are shown in Table 1. Atomic models are built with dimensions: 43×10×26 nm^3^ along the *x* (or $x^{\prime }$ ), *y* (or $y^{\prime }$ ), and *z* (or $z^{\prime }$ ) directions.

Table 2 summarizes the interfacial energies of the three distinct interfaces. In each case, the most stable interface was identified following the steps in our previous work [14].

**Figure 2:**
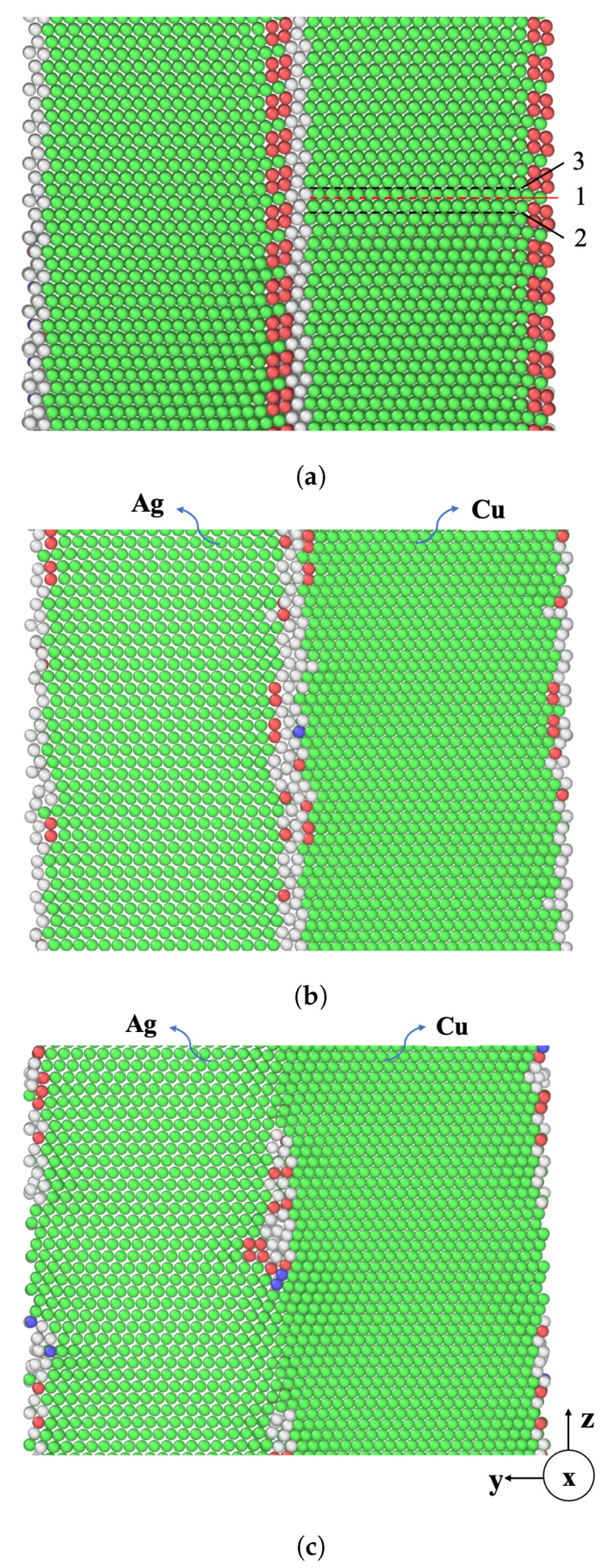
The atomic configurations of (**a**) nanolaminated Ag, (**b**) Ag/Cu type I nanolaminate, and (**c**) Ag/Cu type II nanolaminate. The green, blue, and red colors correspond to FCC, BCC, and HCP atoms, respectively. In (**a**), 1, 2, and 3 are glide planes.

We have constructed five different nanolaminate models, each sharing a uniform individual layer thickness of *L* = 5 nm. The first one is a single-phase nanolaminated Ag system with a {112} SITB and the others are Ag/Cu (type I, type II) with {112} incoherent interfaces. The type I and type II nanolaminates differ in the crystallographic orientations of the Cu layer. In the type II nanolaminates, the orientation relationship between the two layers is also known as the “cube-on-cube” orientation. The nanolaminated Ag SITBs and Ag/Cu (type I, type II) {112} incoherent interface contain a network of misfit (or interfacial) dislocations. Figure 2 illustrates the atomic structures of these interfaces. The Ag/Ag SITB features a repeating arrangement of two parallel sequences, which consist of Burgers vectors ${\vec{b}}_{1}$ and $-2{\vec{b}}_{1}$ on each {111} plane, where ${\vec{b}}_{1}$ represents a Shockley partial dislocation [50].

The numerous parallel glide planes situated within the layers, though sharing a crystallographic equivalence, exhibit distinct relationships with the misfit dislocations found at the layer interfaces. In the case of the SITB within the nanolaminated Ag, the periodic unit encompasses three Ag glide planes, as shown in Figure 2a, with 1, 2, and 3, the same as the nanolaminated Cu [14]. These planes are denoted as Ag1, Ag2, and Ag3, respectively. The calculations for CLS are then repeated for each of these three planes.

Since the incoherent interface of Ag/Cu nanolaminates has a complex structure, it is difficult to determine how many different slip planes there are in each material (i.e., Ag or Cu). Therefore, we simply choose ten adjacent planes in each material and place a dislocation on each plane. Thus, in total, for the interface within each type of Ag/Cu nanolaminate, we study 20 different slip planes. As a result, for the Ag/Cu type I nanolaminate, there are ten {111} planes in which we insert a dislocation designated as Ag1Cu, Ag2Cu, and so forth, as well as ten {111} planes in Cu, referred to as Cu1Ag, Cu2Ag, and so on. Similarly, for the Ag/Cu type II nanolaminate, 20 {111} slip planes are considered in total.

In all cases, we introduce an edge dislocation with a Burgers vector denoted as $\vec{b}$ into grain *M*. Depending on whether the layer under consideration is made up of Ag or Cu, the Burgers vector $\vec{b}$ slightly differs. $\vec{b}=\left(a_{0}/2\right)\langle 110\rangle$ , where $a_{0}$ is the lattice parameter of either Ag (4.09 Å) or Cu (3.615 Å).

## 3. Results and Discussion

### 3.1. Nanolaminated Ag, SC-Ag, and FS-Ag

**Figure 3:**
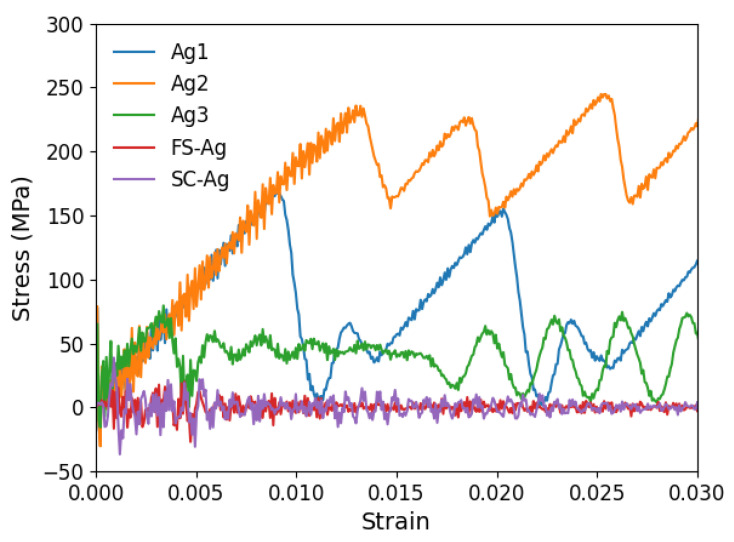
Shear stress–strain curves for the CLS in an Ag layer of a nanolaminated Ag. Responses to dislocation glide on planes Ag1, Ag2, and Ag3 are shown. For comparison, the response to edge dislocation gliding in Ag single crystals is included. SC-Ag is when PBCs are applied along the dislocation line; FC-Ag is when traction-free boundary conditions are applied along the dislocation line.

The shear stress–strain curves depicted in Figure 3 focus on the varying responses of dislocations on glide planes Ag1, Ag2, and Ag3 within nanolaminated Ag, SC-Ag, and FS-Ag. It reveals that the shear stress required to move dislocations is significantly dependent on the specific glide plane. The movement of dislocations is oscillatory across all planes, characterized by more forward than backward motion, particularly evident in the Ag1 and Ag2 planes. The Ag2 curve shows the highest peak stress, suggesting the highest resistance to dislocation glide on this particular plane. Dislocations on all planes begin to move when the first-peak stress is reached, with 170 MPa for Ag1, 234 MPa for Ag2, and 80 MPa for Ag3. The mean value is 161.33 MPa. Once the peaks are reached, a rapid decrease to 5 MPa for Ag1, 155 MPa for Ag2, and near zero for Ag3 is followed, before a subsequent increase. The stress for Ag1, Ag2, and Ag3 drops at strains of 0.009, 0.01335, and 0.003375, respectively. It is worth noting that the Ag3 curve has more frequent oscillations with additional strain, which can be attributed to the intense interactions with defects present at the surrounding SITB. The difference in peak stress is likely to be the result of different locations of the misfit dislocations at the SITB. The interface structures for each plane are shown in Figure 2. There are two planes Ag1 and Ag3, which are above and below plane Ag2, which corresponds to the plane on which the ${\vec{b}}_{1}$ misfit dislocation has extended.

**Figure 4:**
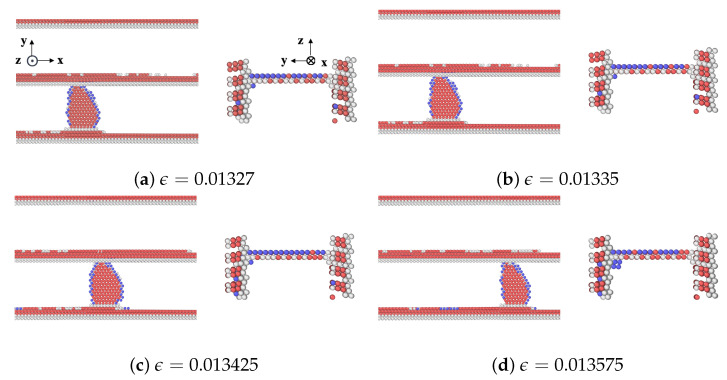
A series of atomic configurations at different shear strains ϵ for the CLS on plane 2 in the nanolaminated Ag. Blue, red, and gray represent BCC, HCP, and unknown coordination structure atoms, respectively. All FCC atoms have been removed to enhance the visibility of interfaces and dislocations. (**b**) Corresponds to the first peak stress in the stress–strain curve.

A detailed analysis of the motion of the edge dislocation on plane Ag2 during the initial stages of straining is provided in Figure 4. With respect to Figure 4a, the strain in Figure 4b has increased slightly, and that is where the dislocation movement begins. This is a critical point in the deformation process where the material begins to yield or deform plastically. As mentioned earlier, the first peak stress of plane Ag2 corresponds to the stress needed for the gliding dislocation to overcome the resistance from the extended misfit dislocation. As the shear strain increases further in Figure 4c, the dislocation has moved or propagated through the nanolaminate. The configuration of atoms changes in response to the different applied strains. The dislocation did not move from one plane to another in the nanolaminated Ag, i.e., no dislocation climb was observed.

**Figure 5:**
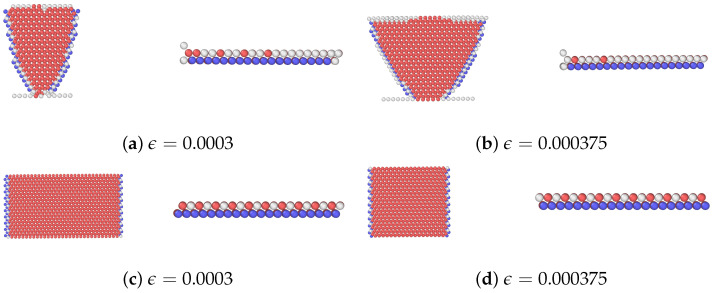
A series of atomic configurations at different shear strains ϵ for the (**a**,**b**) FS-Ag and (**c**,**d**) SC-Ag. Blue, red, and gray represent BCC, HCP, and unknown coordination structure atoms, respectively. All FCC atoms have been removed to enhance the visibility of interfaces and dislocations. (**a**,**c**) correspond to the first peak stress for FS-Ag and SC-Ag, respectively. The coordinates are the same as Figure 4a.

As shown in Figure 3, the shear stress–strain curves for both SC-Ag and FS-Ag have a non-smooth glide process and do not involve backward motion, unlike in the nanolaminated Ag. Notably, within the SITB interface, the periodicity of the occurrence of misfit dislocations matches the periodicity of the lattice structure. However, there is a lack of interaction between the gliding dislocation and misfit dislocations in SC-Ag and FS-Ag. FS-Ag and SC-Ag show similar patterns, with stress levels fluctuating around zero and decreasing as the strain increases. The initial peak stress in both SC-Ag and FS-Ag is significantly lower than in the nanolaminated Ag, being around 4 MPa for SC-Ag and 5.7 MPa for FS-Ag. In a Cu single crystal, it was also found that the SC model has a lower critical stress than the FS model [14]. The primary resistance to dislocation motion in the SC-Ag comes from the lattice itself. Whereas, for FS-Ag, the resistance to dislocation glide is influenced by two main factors: the inherent resistance from the lattice structure and the additional confinement resistance from the surrounding free surfaces. The behavior observed in the SC-Ag and FS-Ag models is characterized by jerky movements, marked by a continuous cycle of rapid acceleration followed by sudden stops or gradual motion. The atomic configurations at the strain level at the first peak stress and that right after that are shown in Figure 5 for SC-Ag and FS-Ag.

### 3.2. Ag/Cu Type I Nanolaminate

**Figure 6:**
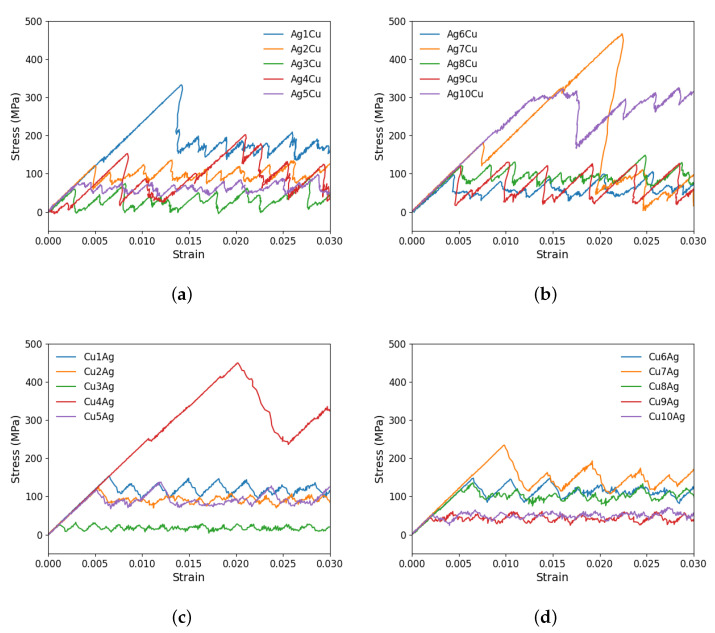
Shear stress–strain curves for the CLS in either an Ag layer or a Cu layer in the Ag/Cu type I nanolaminate. CLS in ten distinct glide planes in (**a**,**b**) the Ag layer and (**c**,**d**) the Cu layer.

According to Figure 6, diverse stress–strain behaviors are observed in the Ag/Cu type I nanolaminate. Initially, as the strain is applied, the stress increases linearly without any dislocation movement. A peak stress for CLS is reached at the point where the dislocation begins to move within the layer, followed by a sharp stress drop and subsequent oscillations. The dislocation movement is not continuous but rather a jerky back-and-forth motion. In each plane, there is a unique, non-linear response associated with varying stress levels. In other words, the glide plane has a significant effect on dislocation behavior. The peak stresses in the ten planes are distinct from one another for both the Ag and Cu layers.

We further investigate the initial movement of dislocations on two specific glide planes. Within the Ag layer, the highest peak stress corresponds to Ag1Cu, 332.69 MPa. Within the Cu layer, Cu4Ag has the highest peak stress value of 449.43 MPa. Compared to other planes, these planes require significantly higher stresses for dislocation movement. Conversely, plane 4 in the Ag layer (i.e., Ag4Cu) and plane 3 in the Cu layer (i.e., Cu3Ag) exhibit the lowest stress levels, being 23.14 MPa and 26.23 MPa, respectively. As shown in Table 3, the mean critical stress in the Ag layer (145.05 MPa) is slightly lower than that in the Cu layer (150.21 MPa). Also, note that the mean critical stress in the Ag layer is lower than that in the nanolaminated Ag, 161.33 MPa.

**Figure 7:**
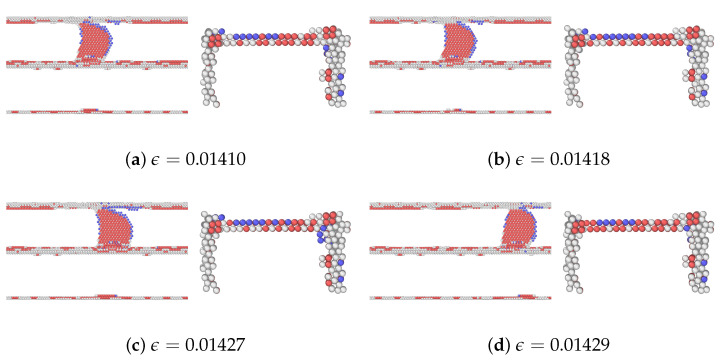
A series of atomic configurations at various shear strains ϵ for the CLS in the case of Ag1Cu in the Ag/Cu type I nanolaminate. Blue, red, and gray represent BCC, HCP, and unknown coordination structure atoms, respectively. All FCC atoms have been removed to enhance the visibility of interfaces and dislocations. (**b**) Corresponds to the first peak stress in the stress–strain curve. The coordinates are the same as Figure 4a.

**Figure 8:**
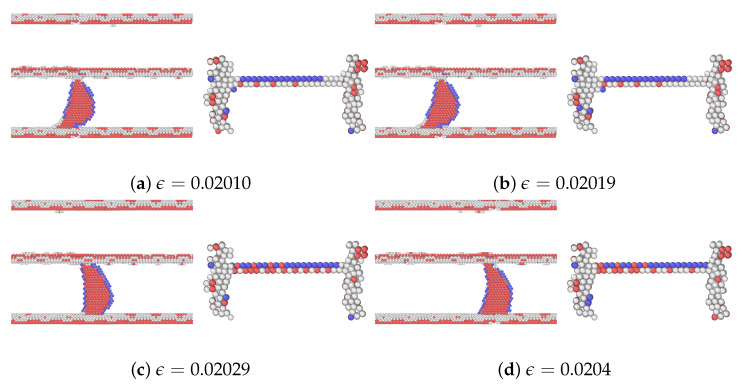
A series of atomic configurations at various shear strains ϵ for the CLS in the case of Cu4Ag in the Ag/Cu type I nanolaminate. Blue, red, and gray represent BCC, HCP, and unknown coordination structure atoms, respectively. All FCC atoms have been removed to enhance the visibility of interfaces and dislocations. (**b**) Corresponds to the first peak stress in the stress–strain curve. The coordinates are the same as Figure 4a.

As mentioned, among the glide planes, Ag1Cu and Cu4Ag exhibit the highest peak stresses when CLS takes place in the two layers, respectively. Figure 7 and Figure 8 demonstrate the atomic configurations of dislocation for plane 1 in Ag and plane 4 in Cu. The dislocation begins to move at a strain of 0.014475 in Ag and 0.02025 in Cu. When the strain is further increased, the dislocation moves further, as shown in Figure 7b and Figure 8b. We also examined the dynamics of dislocations gliding under strain. The first peak stress indicates the stress required to initiate the glide of dislocations, followed by a decrease that facilitates further glide. This pattern of the strain–stress curve is observed across all planes in this glide. After the stress drop, there is an increase in the stress level until another peak is reached at a sufficiently high-stress level to restart CLS. There is no evidence of dislocation climbs in any of the cases.

### 3.3. Ag/Cu Type II Nanolaminate

**Figure 9:**
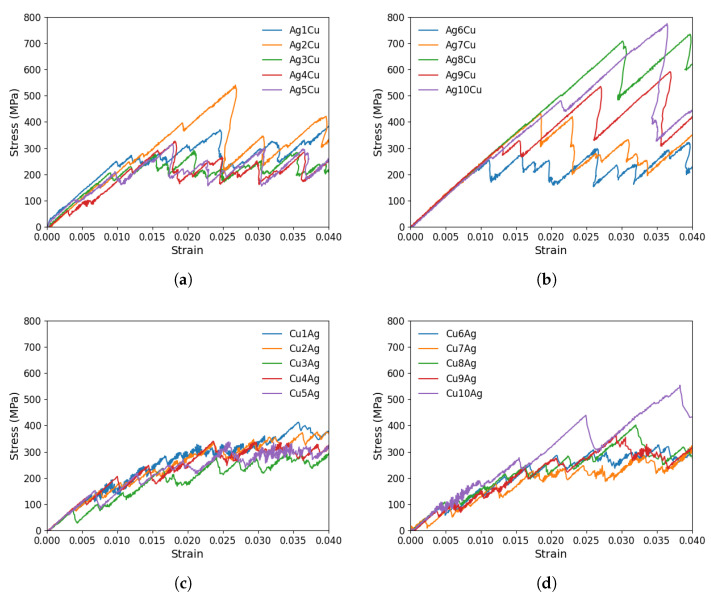
Shear stress–strain curves for the CLS in either an Ag layer or a Cu layer in the Ag/Cu type II nanolaminate. (**a**,**b**) CLS in ten distinct glide planes in the Ag layer. (**c**,**d**) CLS in ten distinct glide planes in the Cu layer.

As shown in Table 1, Cu and Ag layers have the same crystallographic orientations across the interface in the Ag/Cu type II nanolaminate. The relevant stress–strain curves are provided in Figure 9.

There are several peaks and drops in the stress–strain curves. It is evident from these curves that there is a substantial difference between the yield strength of distinct planes. The maximum peak stress is found for plane 8 (Ag8Cu) when CLS occurs in Ag, i.e., 774.46 MPa when the strain is 0.0364. The stress level of most other planes is considerably lower. In the Cu layer, the highest peak stress to start the dislocation motion in this nanolaminate occurs in the glide plane 1 with the stress being 282.50 MPa. Between the two layers, the mean peak stress is much higher in the Ag layer (346.31 MPa) than in the Cu layer (140.12 MPa). Compared with the nanolaminated Ag and Ag/Cu type I nanolaminate, the mean critical stress for CLS in the Ag layer is higher in the Ag/Cu type II nanolaminate. For CLS in the Cu layer, the mean critical stress is higher in the Ag/Cu type I nanolaminate than in the type II one.

**Figure 10:**
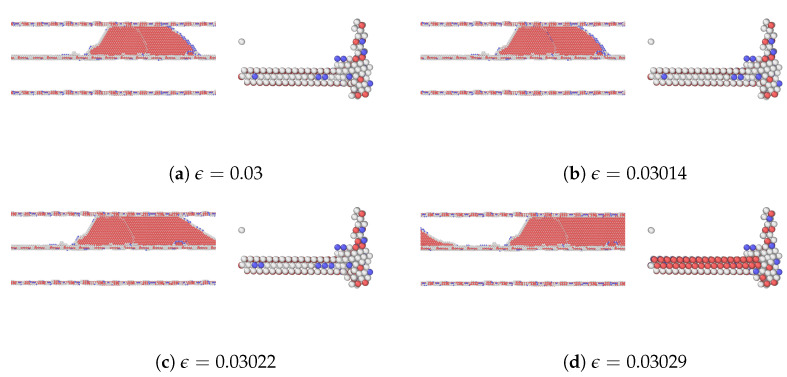
A series of atomic configurations at various shear strains ϵ for the CLS in the case of Ag8Cu in the Ag/Cu type II nanolaminate. Blue, red, and gray represent BCC, HCP, and unknown coordination structure atoms, respectively. All FCC atoms have been removed to enhance the visibility of interfaces and dislocations. (**b**) Corresponds to the first peak stress in the stress–strain curve. The coordinates are the same as Figure 4a.

**Figure 11:**
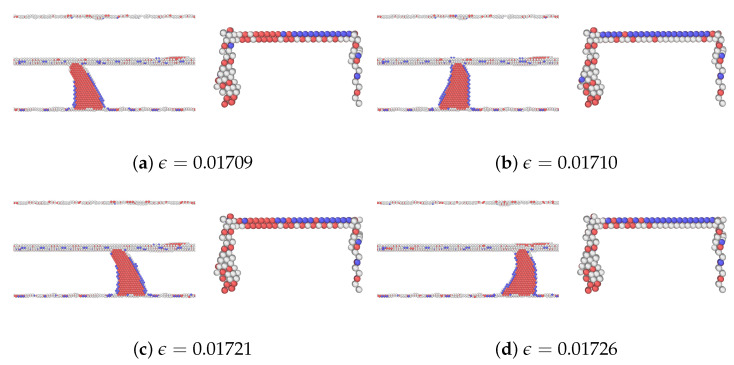
A series of atomic configurations at various shear strains ϵ for the CLS in the case of Cu1Ag in the Ag/Cu type II nanolaminate. Blue, red, and gray represent BCC, HCP, and unknown coordination structure atoms, respectively. All FCC atoms have been removed to enhance the visibility of interfaces and dislocations. (**b**) Corresponds to the first peak stress in the stress–strain curve. The coordinates are the same as Figure 4a.

The atomic configurations corresponding to the movement of dislocation in glide plane 8 in the Ag layer and plane 1 in the Cu layer are shown in Figure 10 and Figure 11, respectively. In the cases of Ag8Cu and Ag10Cu, the dislocation partially climbs in the Ag layer, similar to the finding in a nanolaminated Cu [30]. Unlike the previous work, however, the dislocation was also expanded within the slip plane in Ag10Cu (but not in Ag8Cu), as shown in Figure 10. We note that the Ag10Cu case has the highest peak stress among all glides in the Ag layer, in agreement with the previous findings that dislocation climb tends to occur when the critical stresses for CLS are high [14,30].

## 4. Conclusions

In this study, atomistic simulations were conducted to investigate the influence of interface morphology on CLS in nanolaminated Ag and two types of Ag/Cu nanolaminates. Each nanolaminate is modeled with a 5 nm layer thickness. We found that the structure of the interface plays a critical role in determining both how CLS occurs as well as the level of stress required to initiate it. Our results indicate the following:

- The CLS of the nanolaminated Ag exhibits oscillatory behavior, characterized by the dislocation moving forward and then reversing its direction repeatedly. Dislocations advance more forward than backward, which results in an overall forward advancement as the strain increases.
- We also consider two Ag single crystals, which exhibit substantially lower initial peak stresses than all nanolaminates.
- There are irregular stress–strain responses for CLS in the Ag/Cu nanolaminates, either highly fluctuating or featuring periods of sharp stress increases followed by rapid stress declines. In the Ag/Cu nanolaminates, characterized by an incoherent interface with misfit dislocation arrays, the CLS exhibits jerky behavior in both the Ag and Cu layers. For the Ag layer, the CLS is most difficult to initiate in the Ag/Cu type II nanolaminate, followed by the nanolaminatesd Ag, and the easiest in the Ag/Cu type I nanolaminate. Interestingly, the order is the same as that in their interfacial energies. For the Cu layer, the CLS is more difficult in the type I nanolaminate than the type II one.
- Our study in this paper primarily focuses on unit CLS processes involving a single dislocation and three interfaces. Therefore, our findings cannot be directly used to understand the macroscopic behavior of nanolaminates, which are characterized by an abundance of dislocations and interfaces. Nevertheless, our results might offer insights and serve as a foundational guide for future research on nanolaminates. Additionally, these nanolaminates are sometimes used in constructions exposed to intense irradiation. Such harsh environments gradually lead to the formation and accumulation of defects within the materials, ultimately resulting in internal damage [51,52]. For example, irradiation-induced voids and helium bubbles may evolve into large clusters. Therefore, studying the CLS in these systems becomes crucial and will be in our future work.

## Figures and Tables

**Table 1:** The crystallographic orientations of single crystal layers *M* and $M^{\prime }$ in the nanolaminates systems.

Nanolaminates *(M/M′)*	*x*	*y*	*z*	*x′*	*y′*	*z′*
Ag/Ag	[1 $\bar{1}$ 0]	[11 $\bar{2}$ ]	[111]	[1 $\bar{1}$ 0]	[112]	[ $\bar{1}$ $\bar{1}$ 1]
Ag/Cu type I	[1 $\bar{1}$ 0]	[112]	[ $\bar{1}$ $\bar{1}$ 1]	[1 $\bar{1}$ 0]	[11 $\bar{2}$ ]	[111]
Cu/Ag type I	[1 $\bar{1}$ 0]	[11 $\bar{2}$ ]	[111]	[1 $\bar{1}$ 0]	[112]	[ $\bar{1}$ $\bar{1}$ 1]
Ag/Cu type II	[1 $\bar{1}$ 0]	[11 $\bar{2}$ ]	[111]	[1 $\bar{1}$ 0]	[11 $\bar{2}$ ]	[111]
Cu/Ag type II	[1 $\bar{1}$ 0]	[11 $\bar{2}$ ]	[111]	[1 $\bar{1}$ 0]	[11 $\bar{2}$ ]	[111]

**Table 2:** Interfacial energies of three interfaces in mJ/m^2^.

Ag/Ag	Ag/Cu Type I	Ag/Cu Type II
407.16	580.79	474.74

**Table 3:** The critical stresses for each glide plane, in units of MPa, in two Ag/Cu nanolaminates.

Glide Plane	Ag/Cu Type I	Ag/Cu Type II
Ag1Cu	332.69	370.11
Ag2Cu	121.39	540.56
Ag3Cu	59.84	205.24
Ag4Cu	23.14	327.14
Ag5Cu	77	315.86
Ag6Cu	97.29	254
Ag7Cu	181.17	431.69
Ag8Cu	121.05	707.97
Ag9Cu	120.26	309.63
Ag10Cu	316.64	480.72
Mean value	145.05	346.31
Cu1Ag	153.03	282.50
Cu2Ag	123.81	152.96
Cu3Ag	26.23	76.28
Cu4Ag	449.43	121.82
Cu5Ag	116.50	152.60
Cu6Ag	147.83	93.70
Cu7Ag	234	47.05
Cu8Ag	135.53	121.30
Cu9Ag	61	75.66
Cu10Ag	54.80	277.41
Mean value	150.21	140.12

## Data Availability

The data presented in this study are openly available at https://github.com/shuozhixu/Materials_2024 (accessed on 15 January 2024).
